# Math Anxiety Assessment with the *Abbreviated Math Anxiety Scale:* Applicability and Usefulness: Insights from the Polish Adaptation

**DOI:** 10.3389/fpsyg.2015.01833

**Published:** 2015-11-30

**Authors:** Krzysztof Cipora, Monika Szczygieł, Klaus Willmes, Hans-Christoph Nuerk

**Affiliations:** ^1^Department of Philosophy, Institute of Psychology, Jagiellonian UniversityKraków, Poland; ^2^Copernicus Center for Interdisciplinary StudiesKraków, Poland; ^3^Section Neuropsychology – Department of Neurology, University Clinic RWTH Aachen UniversityAachen, Germany; ^4^Department of Psychology and LEAD Graduate School, University of TuebingenTuebingen, Germany; ^5^Knowledge Media Research Center, IWM-KMRCTuebingen, Germany

**Keywords:** AMAS, math anxiety, anxiety, confirmatory factor analysis, cross-cultural studies, healthy adults

## Abstract

Math anxiety has an important impact on mathematical development and performance. However, although math anxiety is supposed to be a transcultural trait, assessment instruments are scarce and are validated mainly for Western cultures so far. Therefore, we aimed at examining the transcultural generality of math anxiety by a thorough investigation of the validity of math anxiety assessment in Eastern Europe. We investigated the validity and reliability of a Polish adaptation of the *Abbreviated Math Anxiety Scale* (AMAS), known to have very good psychometric characteristics in its original, American-English version as well as in its Italian and Iranian adaptations. We also observed high reliability, both for internal consistency and test-retest stability of the AMAS in the Polish sample. The results also show very good construct, convergent and discriminant validity: The factorial structure in Polish adult participants (*n* = 857) was very similar to the one previously found in other samples; AMAS scores correlated moderately in expected directions with state and trait anxiety, self-assessed math achievement and skill as well temperamental traits of emotional reactivity, briskness, endurance, and perseverance. Average scores obtained by participants as well as gender differences and correlations with external measures were also similar across cultures. Beyond the cultural comparison, we used path model analyses to show that math anxiety relates to math grades and self-competence when controlling for trait anxiety. The current study shows transcultural validity of math anxiety assessment with the AMAS.

## Introduction

### Definition and societal importance

Math underachievement and its broad social and personal consequences attract increasing attention from both scientific investigation and educational policy (e.g., OECD, 2010). It was already known in the 1970's that intelligence accounts for only 50% of the variance in math performance (see Suinn and Edwards, 1982). Math anxiety is considered to be one important factor contributing to individual math achievement. Interestingly, in past decades extensive research on this phenomenon was conducted mostly in the United States and Great Britain. Recently, the math anxiety construct has received more attention in other countries (see, e.g., Krinzinger et al., 2009). Recent developments in studies on math anxiety are reviewed by Suárez-Pellicioni et al. (2015). Nevertheless, we do not know how universal construct validities and psychometric properties of the mostly English studies are, especially as regards Eastern Europe, since such data are as of yet lacking. Therefore, in this study we examined the Polish adaptation of the *Abbreviated Math Anxiety Scale* (AMAS; Hopko et al., 2003).

The term math anxiety refers to negative states related to math and mathematical situations (for definition, history, and consequences see Ashcraft and Ridley, 2005). This very general definition can be expanded so that math anxiety refers to a wide range of negative emotional states that accompany an individual when struggling with math in different situations. These emotional responses vary from apprehension to fear and dread. These situations may involve everyday activities, in which an individual has to deal with numbers (e.g., financial transactions) as well as academic matters. Math anxiety leads to cognitive (e.g., worrisome thoughts) and behavioral (avoidance) consequences (see: Krinzinger et al., 2009 for comparison). Interestingly, Faust (1992; see Ashcraft and Ridley, 2005) claims that math anxiety meets the criteria of genuine phobia. It is therefore widely accepted that math anxiety is different from other forms of anxiety (see Suárez-Pellicioni et al., 2015). The independence of math anxiety from other possibly related constructs will be further discussed.

### Math anxiety and its relations to other cognitive and personality variables

Importantly, math anxiety cannot be reduced to poor math performance, since the differences in math performance between high and low math anxious individuals depend largely on math testing conditions (Ashcraft and Ridley, 2005). Anxiety responses already appear when an individual is expecting to face mathematical problems (Lyons and Beilock, 2012). Therefore, measurement of math achievement itself may be compromised by math anxiety. The relationship between math achievement and math anxiety has been the subject of numerous studies summarized in a meta-analysis by Ma (1999). The average correlation between math anxiety and math grades was −0.27. The association between math anxiety and math performance, however, seems to be limited to areas of math with a strong involvement of numbers (e.g., Vukovic et al., 2013).

Besides performance measures, math anxiety also correlates with a number of personality constructs, providing some insights into its convergent and divergent validity. Ashcraft and Ridley (2005, p. 317, see also Ashcraft and Moore, 2009) give a summary of correlation coefficients between math anxiety and several other variables. It is based on two meta-analyses by Hembree (1990) and Ma (1999). If not stated explicitly, the data are taken from those meta-analyses. Here we discuss only correlations that are relevant for the purpose of the presented study.

In general, math anxiety correlates moderately with other forms of anxiety (*r* ≈ 0.40), apart from test anxiety in which correlations are quite strong. Namely, correlations between several measures of math anxiety are considerably higher than correlations between measures of math anxiety and measures of other types of anxiety, thereby suggesting discriminant validity of the construct. This pattern of correlations is present both in children and in adults. Specific self-concepts (i.e., math self-concept and math self-efficacy) are also related to math anxiety. Math self-efficacy is defined as perceived ability to solve pure and applied math problems, whereas the term math self-concept denotes perceived competence in math (OECD, 2013). The correlations between math self-concept and math self-efficacy are extremely high (often above 0.90) which means that these constructs are hardly distinguishable from each other both practically and theoretically (Lee, 2009 for discussion). Nevertheless, those concepts were distinguished in 33 of 41 countries involved in the PISA study. The relation between these concepts and math achievement is also similar to the relation between math anxiety and math achievement (Lee, 2009).

In sum, math anxiety is correlated both with cognitive measures like math performance as well as with personality measures like state and trait anxiety, test anxiety as well as self-concept and self-efficiency in math.

### Short-term effects of math anxiety

Apart from long-term consequences of math anxiety, several short-term effects have been described. First of all, math anxiety may lead to so-called local avoidance. Highly math anxious individuals, when faced with math problem, feel uncomfortable and want to terminate this anxiety-evoking situation. This often leads to sacrificing accuracy for speed (i.e., random or unchecked answers are given; see Ashcraft and Ridley, 2005). Moreover, several cognitive consequences of math anxiety were described. First of all, anxiety reduces working memory capacity, which leads to decrements in performance (Ashcraft and Krause, 2007; see also Suárez-Pellicioni et al., 2015, for review). This is in line with general claims on how anxiety impairs cognition (see: Eysenck and Calvo, 1992). Solving math problems requires working memory capacities of storing, updating intermediate results and performing calculations. However, recent developments indicate that the relation between anxiety and working memory capacity is more complex. Individuals having less working memory capacity show deficits in inhibiting emotional responses (Hofmann et al., 2012). This aspect is extensively discussed by Trezise and Reeve (2014), who also point out that several patterns of working memory capacity and worry are less stable over time than others. Namely in some individuals, WM capacity and perceived level of worry change over measurements, whereas in other individuals they remain stable over time. Most dynamic changes were observed in individuals, who scored high in worry and in working memory in the initial test, whereas results were most stable in individuals, who scored low in worry and high in working memory capacity. These changes may also be associated with temporal fluctuations in math problem solving performance. Importantly, decrements in performance may be associated with tiredness, even within 1 day. Similarly Chuderski (2015) shows that the relation between WM capacity and anxiety is not present in high fluid intelligence individuals.

In sum, math anxiety is not only related to personality traits and long-term arithmetic skill, but also impairs short-term functioning in mathematical examination situations. However, pattern of these short-term relationships may differ across individuals.

### Gender differences

Female individuals—both adults and children—tend to reveal higher levels of math anxiety. Female individuals having the same cognitive capacities perform worse on math tests because their performance is compromised by math anxiety (Devine et al., 2012). Gender differences in math anxiety were also found in Great Britain (Hunt et al., 2011). On the other hand, Ma (1999) showed that there is no gender difference in the correlation between math anxiety and math achievement. Math anxiety was also shown to be more stable over time in female compared to male individuals. On the other hand, in male individuals, the relationship between past math achievement and later math anxiety is more pronounced (Ma and Xu, 2004). It is important to note that there are also studies reporting no gender differences or even higher Math anxiety in male participants (see: Devine et al., 2012 for a short review). Results of PISA 2012 study (OECD, 2013) show that in vast majority of OECD countries, averaged effect size of gender difference is small but meaningful (*d* = 0.30). In Polish adolescents the effect size can be considered as very small (*d* = 0.11). Similar differences were also observed in case of related constructs, namely math self-efficacy and math self-concept. As regards math self-efficacy, effect size averaged across OECD countries was 0.34 in favor of boys. This gender difference in Polish adolescents was very small (*d* = 0.14). In case of math self-concept the averaged effect size was 0.36. Again in Poland it was smaller but this time meaningful (*d* = 0.22).

In sum, gender differences in math anxiety as well as other, related concepts are present in most cultures. Female individuals (both adolescents and adults) report stronger math anxiety. Furthermore, they feel less confident when struggling with math problems. These differences are also present in Polish adolescents, however its size is rather small.

### Cultural differences and similarities

The vast majority of research on math anxiety was conducted in the US. Nevertheless, some studies from other countries (mostly Western European and Eastern Asia) are also available. The meta-analysis performed by Ma (1999) suggests that there are no substantial cross-cultural differences in math anxiety. Nevertheless, several individual studies indicate such differences. E.g., Engelhard (1990) shows that Thai students reveal lower levels of math anxiety than their American peers.

Although the amount of research conducted outside the US is relatively small, more recent studies suggest that cultural differences are rather small or non-existent. Math anxiety was reliably measured in Britain (Hunt et al., 2011) as well. The questionnaire used in the study, the *Mathematics Anxiety Scale-UK* (MAS-UK) was adapted from the American original (MAS) to British conditions by eliminating items that were not easily understood by British English speakers. Several items were also added which refer to popular usage of math in British everyday life (e.g., playing darts). The structure of math anxiety was similar in the UK and in the US. Wood et al. (2012) also observed that the structure of math anxiety in school children (second and third graders) was the same in German and Brazilian samples. This result is particularly interesting, since the data come from two very different cultures. Moreover, German and Brazilian children differed considerably in math achievement as measured in the PISA program. Finally, in a study by Ho et al. (2000) the structure of math anxiety was found to be similar in American, Chinese and Taiwanese students. In this study, the two-componential structure of math anxiety was investigated (affective and cognitive aspects). The affective component seems to be consistently related to math achievement. The relations between the cognitive component of math anxiety and math achievement are more inconsistent across cultures.

One large-scale attempt to evaluate math anxiety across different countries was undertaken for data collected in the PISA 2003 program (Lee, 2009). The data was collected in 41 countries. The correlations between the PISA math score and math anxiety varied from about −0.50 (in Denmark, Norway and Poland) to about −0.15 (in Japan, Thailand and Indonesia). In most cases the correlation varied between −0.3 and −0.4 (Lee, 2009, see Table 7 there). Nevertheless, the math anxiety measure was established by means of a factor analysis of the PISA questionnaire data itself. It eventually comprised responses to five items referring to (1) getting nervous when solving mathematical problems; (2) tension when doing math homework; (3) worry that math classes will be too difficult; (4) worry of getting poor math grades; (5) thinking of not being good in math. These items do not allow for the investigation of the structure of math anxiety and responses to some of them (e.g., worry of getting poor grades) may strongly depend on the cultural context. PISA 2003 showed that the correlation between math anxiety and performance in Poland was one of the highest in all countries involved in the programme (*r* = −0.49; Lee, 2009).

The recent PISA 2012 study (OECD, 2013) provided more insights into math anxiety and its relation to math scores and characteristics of math anxiety in Poland. First of all, the relationship between math anxiety and math performance did not change considerably and remained one of the strongest. However, Polish students scored slightly above PISA average in math and slightly below PISA average in math anxiety.

In summary, there were some, yet rather small differences between cultures. However, instruments differed between studies and sometimes (e.g., Hunt et al., 2011) instruments were even changed to adapt them to a certain culture. While this is understandable, it makes cross-cultural comparisons more difficult. Therefore, we will use the same assessment tools as previously examined in the US, Iran and Italy. This allows for a more direct comparison between these countries and Poland.

### Is math anxiety a homogenous construct? structure of math anxiety

So far, in this introduction, we have treated math anxiety as a homogenous construct. However, in general, it is claimed that there are at least two broad components of math anxiety, referring to the use of math in everyday life situations and being tested in math (e.g., Hopko, 2003). This two-factor structure was already proposed in research starting in the early 1970's (see: Suinn and Edwards, 1982). However, 3-factor structures (Alexander and Martray, 1989; Hunt et al., 2011) have also been proposed.

Importantly, there are also approaches that still assume a uni-dimensional structure of Math anxiety. Ashcraft (2002) claims that asking a single question on how math anxious an individual is, may be also a valuable way of math anxiety assessment (the results strongly correlate with results of psychometrically validated math anxiety measurement instruments). A similar approach was also taken in the PISA 2003 study (see Lee, 2009). Núñez-Peña et al. (2014) systematically tested the possibility of assessing math anxiety by using a single item measure called *Single Item Math Anxiety Scale* (SIMA). This instrument is characterized by satisfactory psychometric properties and seems to be an interesting alternative to longer math anxiety assessment instruments.

In sum, the factorial structure of math anxiety is still under debate and differs from author to author.

### The abbreviated math anxiety scale (AMAS)

One of the most interesting instruments for investigating math anxiety, which will also be used in this study, was developed by Hopko et al. (2003). The AMAS is a nine-item questionnaire characterized by very good psychometric properties. The authors present a thorough psychometric evaluation of the AMAS, examining internal consistency, test-retest reliability and several validity measures.

Similar to previous scales, the AMAS total score is composed of two components (1) anxiety related to learning math (*Learning*) and (2) anxiety related to being tested in math (*Testing*). In the presented paper we focus on this questionnaire because of several reasons. First, the short form together with its very good psychometric properties makes it a very good tool for further research. It is suitable for testing both adults and school children (aged 11–16; Devine et al., 2012). Second, the administration of the AMAS takes <5 min and therefore, apart from studies focusing directly on math anxiety, it can easily be included in studies on numerical cognition.

The AMAS was successfully adapted to cultures largely differing from the US. Vahedi and Farrokhi (2011) studied the Iranian adaptation of AMAS, whereas Primi et al. (2014) presented its Italian adaptation. Both studies provided further evidence for the construct validity and reliability of this assessment tool. Results of these studies suggest that the AMAS is suitable for testing math anxiety in varied cultural and linguistic contexts. Furthermore, the factor structure of the AMAS remains invariant and did not show gender differences. Gyuris and Everingham (2011) administered a modified AMAS to Australian students. In the modified version two items about dealing with graphs were added and the item about the pop-quiz was modified stating that the quiz was not for credit. In general, the pattern of results followed the results obtained in the US study; nevertheless modifications introduced by the authors prevent direct comparisons. Convergent and discriminant validity of the AMAS was established by correlating its results with other math anxiety measures (e.g., sMARS; Hopko et al., 2003); test anxiety (e.g., TAI; Hopko et al., 2003; Primi et al., 2014); state and trait anxiety (e.g., STAI; Hopko et al., 2003); math attitudes, motivation to learn, etc… (Vahedi and Farrokhi, 2011; Primi et al., 2014); math grades (Gyuris et al., 2012). All these analyses revealed satisfactory validity indices. However, no measures of attitudes toward humanities were tested (as an indicator of discriminant validity). Furthermore, to the best of our knowledge, no measures of general personality/temperament were used in studies examining psychometric properties of the AMAS scale.

Properties of AMAS scale we reported above make it very useful math anxiety assessment tool for studies on numerical cognition. This is particularly important since sources of individual differences in several aspects of numerical processing are largely unknown (e.g., Cipora and Nuerk, 2013; Hoffmann et al., 2014a,b). There is recent evidence indicating a relationship between math anxiety and elementary numerical processing (e.g., Maloney et al., 2011). In some of those studies, the authors explicitly call for the inclusion of math anxiety as a covariate in studies on numerical cognition (Hoffmann et al., 2014b). The AMAS was already used in order to measure math anxiety in studies by Maloney et al. (2010), Maloney et al. (2011), Maloney et al. (2012), Hopko et al. (2005), and Devine et al. (2012). Furthermore, Maloney (2011) in her dissertation reports results of testing a large sample of college students (over 2000) with AMAS and providing further evidence for high reliability of the AMAS. The original version of the AMAS is freely available for research use from Derek Hopko's website.

### Aim of the present study

In the present study, we aimed to investigate possible cultural differences and gender differences in math anxiety level and structure. In particular, we aimed to further evaluate the psychometric properties of the AMAS. The items of this questionnaire were in our opinion applicable to math-learning situations in Poland (so that in our opinion their content did not require changes as was necessary e.g., in the British adaptation of the US-American *Mathematics Anxiety Scale*; Hunt et al., 2011). However, principal applicability does not imply psychometric properties are the same across cultures—construct validity of the Big Five items for instance differs between cultures. Therefore, we focused on examining construct validity, reliability and both convergent and discriminant validity of the AMAS. Moreover, we compared results from a large-scale Polish sample to results described for the US and other countries mentioned above. Based on previous research with some other instruments, we expected that a similar pattern of results would be obtained for convergent and discriminant validity, as was presented in Hopko et al. (2003), as well as obtained in previous studies using other math anxiety measures (i.e., studies summarized by Ashcraft and Ridley, 2005). We also aimed at checking aspects of discriminant validity, assessing whether AMAS scores do not simply reflect general negative attitudes in the school environment.

Since there is no obvious reason to assume otherwise, we expected that the results for the AMAS obtained in the Polish sample would be similar to those obtained in samples from other linguistic and cultural backgrounds. This includes similarities in psychometric properties as well as average scores, but gender differences in that female individuals should exhibit higher math anxiety.

## Methods

### Participants

Eight hundred and fifty-seven participants took part in the study. Six hundred and eighty-eight of them were female, 160 male and nine did not report their gender. Mean age was 21.6 (SD = 4.1) years and ranged from 18 to 49 years (based on information reported by 841 participants). Participants were students from six Polish universities located in three cities (Kraków, Wrocław, and Nowy Sącz). They studied in a wide range of faculties (psychology, education, law, philosophy, Polish literature, English literature, management and production engineering, medical physics, econometrics). Participation was voluntary. Assessment was done during university classes. The study was conducted in accordance with ethical standards of Jagiellonian Univerisity's Institute of Psychology. According to these regulations conducting questionnaire studies at the time the data was collected, explicit consent of Ethics Committee was not required. Questionnaires were distributed across the audience and the students were free to fill it or refrain from filling them (as well as not responding to questions they wished not to respond).

### Materials

#### Math-related measures

##### AMAS

Two trained psychologists (one of them was the first author) whose native language was Polish translated the original AMAS items from English into Polish independently from each other. Subsequently, the final Polish version of the item wordings was established after discussion between the first author (the first translator) and the second author (both of them are native Polish speakers). Thereafter, a trained psychologist, who was not familiar with the original items before, back translated items. Back-translated items were identical to the original ones except for one phrase (*pop quiz*) that was translated correctly semantically, but was not literally identical (*unannounced test*). Therefore, it was concluded that the translation was satisfactory.

The instructions were prepared in Polish as well. They stated that the participant will see some statements below which are related to learning math. He or she was asked to mark next to each statement the level of anxiety it evokes/would evoke in her/him. Similarly as in the original version, a 5-point Likert scale was used. Henceforth the theoretical range of AMAS score is from 9 to 45, in *Learning* scale it is from 5 to 25 and for *Testing* scale from 4 to 20. To make the AMAS consistent with the MAAA scale (described below), only low and high extremes were labeled (*mild anxiety* and *strong anxiety*, respectively). The AMAS was printed on DinA5 (148 × 210 mm) white paper sheets.

##### Math ability, achievement and attitudes (MAAA)

This scale was developed for the purpose of this study. It was comprised of five parts. In the first part, participants were asked to assess their math ability on a 10-point Likert scale. There were four items on this scale (math in general, arithmetic, geometry and solving real life problems). The extremes of the scale were labeled *very bad* and *very good*, respectively.

In the second part, the participants were asked to mark their typical math grades. There were three items, each referring to one of the stages of obligatory education in Poland (1) elementary school (grades 1–6); (2) so called “gymnasium” (grades 7–9); (3) high school (grades 10–12/13 depending on high school type). At all these levels, math classes are an obligatory part of the curriculum. The participants used a scale compatible with the Polish grading system (i.e., from 1 to 6; 1 refers to the worst grade, 6 to the best grade). The extremes of the scale were marked with Polish verbal labels referring to the worst and the best mark respectively.

In the third part there were two items in which the participants marked how fast they get discouraged while solving a mathematical problem and when they have to write a difficult essay in humanities. The answers were again given using a 10-point Likert scale. The extremes (1 and 10) were marked with labels *I get discouraged very fast* and *I am very persistent*, respectively. In the fourth part with two items, the participants had to mark, how often they used some forbidden aid, while struggling with math problems and problems involving humanities. Similarly, the answers were given on a 10-point Likert scale with the extremes 1 and 10 labeled with *very often* and *I always work on my own*, respectively. In the fifth part comprising three items, participants marked how much they liked math, science and humanities. Responses were given using a 10-point Likert scale again with the extremes 1 and 10 marked with *I dislike very much* and *I like very much*, respectively. The MAAA was printed on a DinA4 sheet.

#### General measures

##### Anxiety assessment

The Polish version of the State and Trait Anxiety Inventory (STAI) was used (Spielberger et al., 1970; Polish adaptation by Spielberger et al., 1987) to measure the level of state and trait anxiety. Reliabilities for the age groups 21–40 and 41–54 years, which are relevant for our sample characteristics, were 0.89–0.92 for STAI-X1 (state) and 0.82–0.90 for STAI-X2 (trait), depending on age group and gender (numerically lower reliabilities were found in the male group).

##### Temperament assessment

The *Formal Characteristics of Behavior—Temperament Inventory* (FCB-TI; Strelau and Zawadzki, 1993, 1995) questionnaire is based on the regulative theory of temperament by Strelau, who defines temperament as the “Expression of Energy Level and Temporal Features of the behavior” (Strelau, 2000, p. 164). The FCB-TI is comprised of 120 items with a *YES* and *NO* response format and assesses six temperament traits: “(1) Briskness (BR): tendency to react quickly, to keep a high tempo of performing activities, and to shift easily in responses to changes in the surroundings from one behavior or reaction to another. (2) Perseverance (PE): tendency to continue and to repeat behavior after cessation of the stimuli (situations) evoking the behavior. (3) Sensory Sensitivity (SS): ability to react to sensory stimuli of low stimulative value. (4) Emotional Reactivity (ER): tendency to react intensively to emotion generating stimuli, expressed in high emotional sensitivity and in low emotional endurance. (5) Endurance (EN): ability to react adequately in situations demanding long-lasting or high stimulative activity and under intensive external stimulation. (6) Activity (AC): tendency to undertake behavior of high stimulative value or to supply, by means of behavior, strong stimulation from the surroundings.” (Strelau and Zawadzki, 1995, p. 208). The FCB-TI has high validity. For instance, ER correlates (≈0.7) with neuroticism, and negatively (≈ −0.3) with extraversion; PE correlates with neuroticism as well (≈0.6). BR correlates with extraversion (≈0.3), and negatively with neuroticism (≈ −0.4); EN correlates negatively with neuroticism (≈ −0.5) and positively with extraversion (≈0.2), all measured with Eysenck's EPQ-R questionnaire. Several other validity measures were reported by Strelau and Zawadzki (1995). The scales were also characterized by satisfactory reliabilities as measured with Cronbach alpha (BR = 0.77; PE = 0.79; SS = 0.73; ER = 0.82; EN = 0.85; AC = 0.84).

### Design of the study

#### AMAS reliability

In the presented study we aimed at checking basic psychometric properties of the AMAS. Reliability of the AMAS was assessed in two ways.

First, we used Cronbach alpha as a measure of internal consistency. It was calculated both for the global AMAS score as well as for the scales proposed in the original paper by Hopko et al. (2003).

Despite great popularity in psychometrics, feasibility of the Cronbach alpha coefficient for estimating reliability of Likert type response data has been challenged. Cronbach alpha uses the inter-item correlation matrix in order to obtain an estimate of reliability. The Pearson correlation coefficient may be deflated when the assumption of continuity of the data is violated. This is the case in Likert-type responses. This leads to underestimates of reliability, especially when the response scale is short. Underestimation of reliability is even more severe when scales are comprised of a relatively small number of items (Yang and Green, 2011). Also, non-normal distributions of both true scores and error scores were shown to cause problems with the traditional alpha coefficient (Sheng and Sheng, 2012).

Using polychoric correlation instead of Pearson correlation in order to calculate the alpha coefficient is suggested as an alternative that takes into account that the observed data are not continuous *per se*, but are ordinal manifestations of a continuous latent construct of interest (Zumbo et al., 2007).

Second, we used the test-retest method (by means of Pearson correlations and intraclass correlations). A subsample of 110 participants (only psychology students) filled in the AMAS for a second time 4 months after the first administration. Both the global score and the subscales were analyzed.

#### Construct and scale validity

We assessed the validity of the AMAS in several ways. First, to examine whether the factor structure of the Polish version resembles the original AMAS, a confirmatory factor analysis was carried out. Additionally, an exploratory factor analysis was conducted (see Data Sheet 1). Additionally we conducted exploratory factor analysis for female and male participants separately to investigate whether the factor structure differs between genders. To establish convergent and divergent validity, we used several other measures: anxiety scales, MAAA items referring to math and the Emotional Reactivity scale of FCB-TI for convergent validity; the MAAA items referring to humanities, in order to demonstrate that the AMAS score does not reflect a general negative attitude toward school, as well as being easily discouraged or looking for external help when facing difficult problems, for discriminant validity. No direct predictions were drawn as regards other FCB-TI scales.

Differences in correlations of the AMAS score with other related measures, for which predictions had been derived, were tested for significance by comparing dependent correlation coefficients (Chen and Popovich, 2002).

### Procedure

The data were collected in a group setting, mostly during lectures or seminars. The order of the questionnaires was as follows: AMAS, STAI (state scale first), MAAA scale and finally, FCB-TI. The sessions usually did not exceed 20 min, except for the sessions with the FCB-TI, because the temperament assessment took about 15 additional minutes. A short verbal instruction was given at the beginning. The non-obligatory character of the study was stressed. Participants were informed that anonymized data would be used for scientific purposes only. Participants were asked to read all instructions carefully. Not all participants were administered the state anxiety questionnaire as well as the FCB-TI. As mentioned above, questionnaires were administered during university lectures and seminars and therefore session time was constrained. For that reason we did not administer the FCB-TI to all participants. Temperamental traits measured with the FCB-TI are not supposed to be directly related to math anxiety. We decided to include state anxiety during the course of data collection, which—also because of time constraints—was not included from the onset of data collection. After the questionnaires were collected, a short debriefing was provided, explaining that we aimed to prepare a Polish version of the Math Anxiety questionnaire AMAS.

## Results

### AMAS descriptive statistics

The average *AMAS total* score was 21.9 (SD = 6.6). The average score of the *Learning* scale was 8.3 (SD = 3.7), while for the *Testing* scale it was 13.6 (SD = 4.0). Average scores of the individual items are presented in Table 1. The total scores for the *Testing* and *Learning* scales were moderately correlated (0.49). Both scales strongly correlated with the total score (0.88 and 0.85 for *Testing* and *Learning* scales, respectively). In the *AMAS total* score female participants obtained significantly higher scores than male participants [*t*_(846)_ = 6.64; *p* < 0.001; *d* = 0.61]. Means for female and male participants were 22.6 (SD = 6.6) and 18.9 (SD = 6.7), respectively. Significant differences were present for both scales. For the *Learning* scale mean scores were 8.5 (SD = 3.7) and 7.6 (SD = 3.1) for female and male participants, respectively [*t*_(846)_ = 2.75; *p* = 0.002; *d* = 0.25]. For the *Testing* scale mean scores were 14.2 (SD = 3.9) and 11.3 (SD = 3.9) for female and male participants, respectively [*t*_(846)_ = 8.55; *p* < 0.001; *d* = 0.75].

**Table 1:** **Item analysis of the Polish adaptation of the AMAS questionnaire**.

**Item**	**Item description**	**Sub-scale**	**Descriptive statistics**		Corrected item-total correlations^*^			**CFA, squared multiple correlation**	
			**Mean score**	***SD***	**Total**	**Learning**	**Testing**	**Learning**	**Testing**
1	Using tables	*L*	1.54	0.95	0.53	0.62	0.30	0.16	–
2	Test 1 day before	*T*	3.24	1.29	0.77	0.44	0.87	–	0.68
3	Watching teacher's work	*L*	1.64	0.96	0.66	0.76	0.39	0.36	–
4	Math exam	*T*	3.81	1.18	0.67	0.28	0.84	–	0.60
5	Homework	*T*	2.77	1.21	0.75	0.51	0.77	–	0.53
6	Attending lecture	*L*	1.71	1.07	0.65	0.80	0.35	0.49	–
7	Other student explaining Math	*L*	1.68	0.99	0.58	0.76	0.28	0.42	–
8	Pop quiz	*T*	3.79	1.19	0.71	0.38	0.82	–	0.59
9	New chapter	*L*	1.75	1.04	0.69	0.72	0.48	0.50	–
Sum								1.51	2.40

*The table includes descriptive statistics together with item-total correlations and squared multiple correlations from the confirmatory factor analysis*.

* *p < 0.001*.

We also tested whether AMAS scores differed between students who had math in their current curricula (math group; *n* = 168) and those who did not (non-math group; *n* = 689). As expected, the non-math group scored higher on the AMAS than the math group. For the *AMAS total* scores were 22.4 (SD = 6.8) and 19.8 (SD = 5.3) and the difference was significant [*t*_(855)_ = 4.62; *p* < 0.001; *d* = 0.42]. The difference was also significant for the *Learning* scale [*t*_(855)_ = 3.50; *p* < 0.001; *d* = 0.32] with means 8.5 (SD = 3.8) and 7.4 (SD = 3.0) respectively and for the *Testing* scale [*t*_(855)_ = 4.42; *p* < 0.001; *d* = 0.39] with means 13.9 (SD = 4.0) and 12.4 (SD = 3.6), respectively.

**Figure 1:**
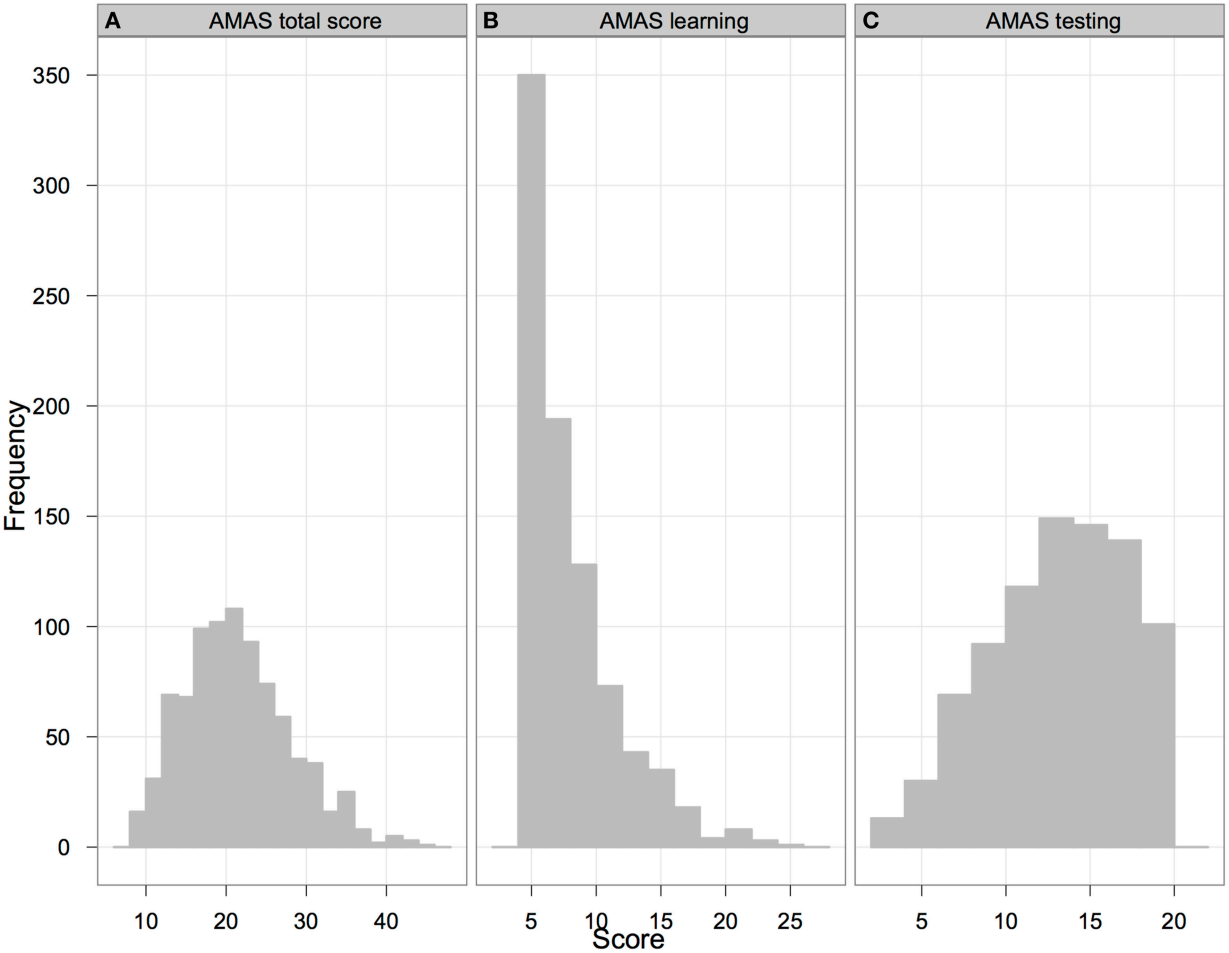
**Distribution of ***AMAS total*** (panel A) and scale totals (panels B and C for Learning and Testing scales respectively)**. The score-range for the *AMAS total* is from 9 to 45, for the *Learning* scale from 5 to 25, for the *Testing* scale from 4 to 20.

The distributions of results for the total score and the subscales are presented in Figures 1A–C. As can be seen in Figure 1A, the *AMAS total* score was close to a normal distribution (skewness = 0.54, SE = 0.08; kurtosis = 0.12, SE = 0.17; both estimates fall within ±2 range so that they can be considered as acceptable; George and Mallery, 2010), but the formal test (*Shapiro-Wilk*_857_ = 0.98; *p* < 0.001) indicated significant deviation from normality. The average score was slightly below the scale midpoint (which is 27). The *Learning* scale was strongly skewed (skewness = 1.51, SE = 0.08; kurtosis = 2.40, SE = 0.17; therefore especially skewness falls outside acceptable ±2 range). A formal test also indicated that the distribution deviated significantly from normality (*Shapiro-Wilk*_857_ = 0.83; *p* < 0.001). Over 230 participants achieved the minimal score, and the average score was substantially below the scale midpoint (which is 15). The distribution of the results of the *Testing* scale was closer to a normal distribution (skewness = −0.34, SE = 0.08; kurtosis = −0.70, SE = 0.17, with both estimates falling within acceptable ±2 range), with the average score close to the scale midpoint of 12. Nevertheless, the formal test again showed a significant deviation from a normal distribution (*Shapiro-Wilk*_857_ = 0.97; *p* < 0.001).

### AMAS reliability

#### Cronbach alpha

First, internal consistency was estimated using the Cronbach alpha coefficient. The reliability estimate was 0.85, 0.78, and 0.84 for the *AMAS total*, the *Learning* scale and the *Testing* scale, respectively. The average inter-item correlation was 0.38, 0.42, and 0.57 for *AMAS total, Learning* scale, and *Testing* scale, respectively. The corrected item-total correlations with the total score as well as with total score for each scale are presented in Table 1.

Additionally, we checked whether there were considerable differences in reliability between mat and non-math groups. The *AMAS total* reliability for the non-math group was 0.85, whereas for the math group it was 0.79. Reliability for the *Learning* scale was 0.79 and 0.70 for the non-math and math groups, respectively. For the *Testing* scale the coefficients were 0.85 and 0.81, respectively.

#### Ordinal alpha

To further explore the reliability of the AMAS, we additionally calculated ordinal Alpha coefficients using the procedure suggested by Gadermann et al. (2012). Ordinal alpha for the AMAS *total* scale was 0.88 for the *Learning* scale 0.84, and for the *Testing* scale 0.87. Ordinal alpha did not increase if any item was dropped; the only exception was an increase by 0.01 in the *Testing* score when item 5—*homework*—was dropped.

#### Test retest reliability

Subsequently, AMAS test-retest reliability was examined by administration of the AMAS to a subsample of 110 psychology students 4 months after the initial testing. First, we compared means and variances in the initial testing of the retest subsample to other participants not taking the retest. Levene's test was used to check for variance equality. The difference in means was significant for the *AMAS total* 20.6 (SD = 5.6) and 22.1 (SD = 6.8) in the retest subsample and other participants respectively, *t*_(855)_ = 2.15; *p* = 0.032; *d* = 0.23. Variances did not differ between groups (Levene's test, *F* = 2.63; *p* = 0.106). The difference in mean *Learning* scale performance was significant as well, 7.3 (SD = 2.6) and 8.5 (SD = 3.8) for the retest subsample and other participants, respectively, *t*_(855)_ = 4.17; *p* < 0.001; *d* = 0.36. Variances differed as well (Levene's test, *F* = 16.97; *p* < 0.001). Contrarily, for the *Testing* scale the mean score did not differ between retest sample (13.4; SD = 3.8) and other participants (13.6; SD = 4.0); *t*_(855)_ = 0.65; *p* = 0.515; *d* = 0.07. There was no difference in variances (Levene's test, *F* = 0.55; *p* = 0.457).

Retest scores for *AMAS total, Learning* and *Testin*g scales were 21.0 (SD = 5.3); 7.8 (SD = 2.5); and 13.2 (SD = 3.7), respectively. The differences in scores for *AMAS total* and *Testing* scale were not significant between the initial testing and the retest (*p'*s > 0.45). For the *Learning* scale, the difference was significant [*t*_(109)_ = −2.05; *p* = 0.042].

Subsequently, test-retest reliabilities were estimated via Pearson correlations. These reliabilities were: 0.71, 0.59, 0.71 for the *AMAS total, Learning* scale, and *Testing* scale, respectively.

The observed floor effect in *Learning* scale as well as the lower variability in this scale in the retest subsample in the initial testing, most probably account for poor test-retest reliability of the *Learning* scale. Furthermore, a significant difference in *Learning* scale between the initial testing and the retest indicate that this reliability estimate must be taken with caution.

Pearson correlation is an estimate of test-retest reliability if two measurements are essentially tau-equivalent (i.e., the variance is identical and the true scores change only in a constant value that is identical for all participants (Ludbrook, 2002; Weir, 2005). Therefore, we additionally computed intraclass correlations (ICC) that take into account consistency of performance from test to retest and change in average performance of participants as a group over time (i.e., change in mean; Vaz et al., 2013). It is therefore more suited here since we observed significant difference in *Learning* scale between test and retest. We used the two-way random effects model with absolute agreement. In all instances ICCs (for single measures) were identical to the above Pearson correlations for the first two decimals. Therefore, differences in *Learning* scale were not substantial.

### Factor structure–confirmatory factor analysis

The presented version of the AMAS questionnaire was an adaptation of an already established scale. Therefore, construct validity was analyzed by means of confirmatory factor analysis. We aimed at testing the structure of math anxiety and its components using structural equation modeling. The model was built in such a way that it matched the original factor structure of the AMAS (also found in an exploratory factor analysis—Data Sheet 1). It involved two correlated latent variables representing the *Learning* and *Testing* Math anxiety components. Items 1, 3, 6, 7, 9 were assumed to contribute to the *Learning* latent variable, whereas items 2, 4, 5, 8 were assumed to contribute to the *Testing* latent variable. We found that the multivariate normality assumption was violated (multivariate kurtosis = 25.90 < critical ratio = 26.94); therefore an asymptotically distribution-free (ADF) method was used. For the same reason, CMIN/DF measures of model fit are not reported, since they are sensitive to violations of the normality assumption (Bedyńska and Książek, 2012).

**Figure 2:**
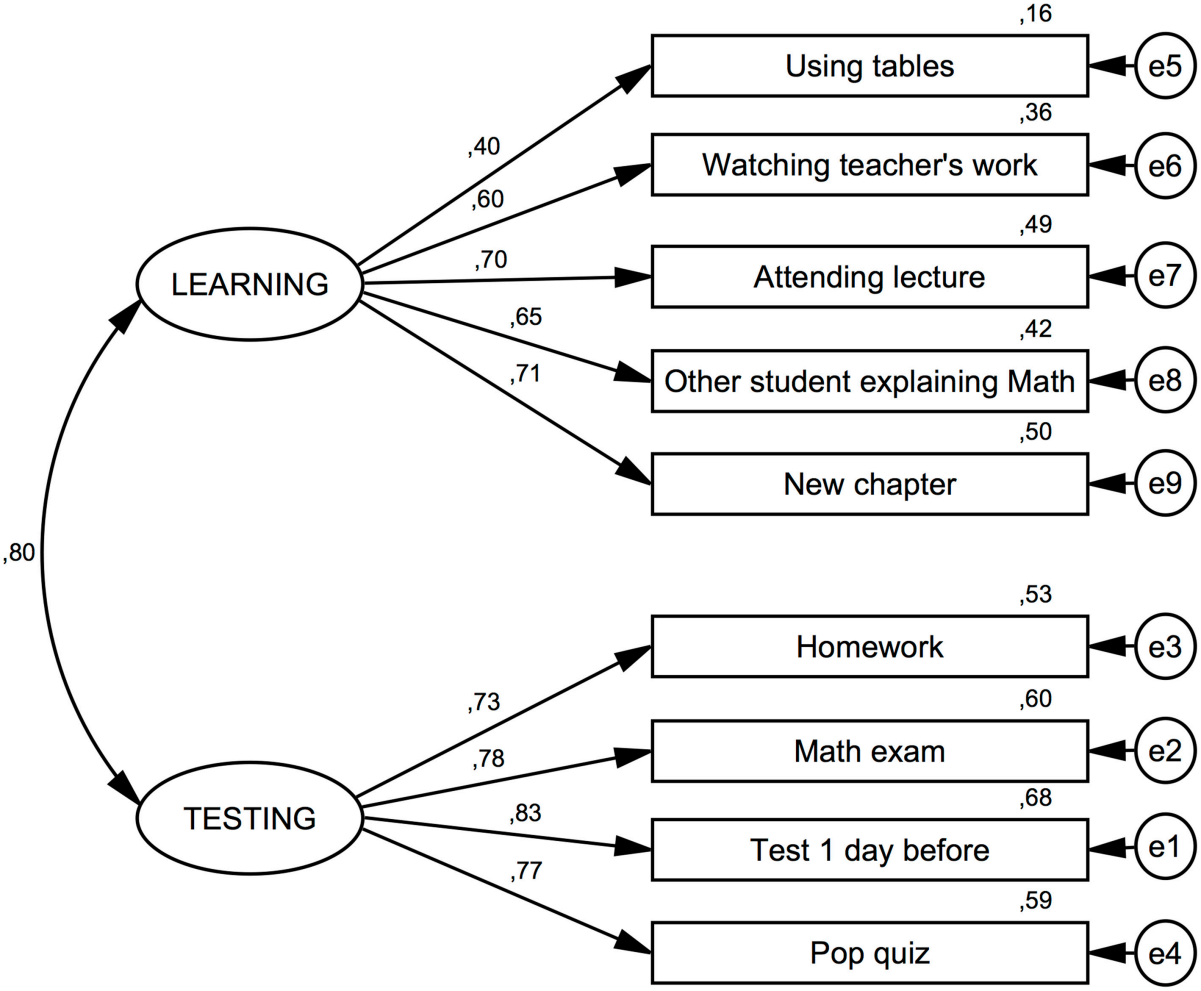
**Confirmatory Factor Analysis for the AMAS**. Indices of model fit are provided and discussed in the main text. The results of the confirmatory factor analysis show that the internal structure of the Polish adaptation of the AMAS is similar to the structure found in the US-American sample. Standardized coefficients are provided for the structural equation model. Variables labeled with e1, e2 etc…denote the respective error terms.

The model, together with standardized path coefficients, is presented in Figure 2. All parameter estimates were found to be significantly different from zero.

As can be seen in Figure 2, apart from Item 1 (*using tables*), all loadings are at acceptable levels (>0.60). Squared multiple correlations between items and the respective subscales are reported in Table 1. Apart from Item 1 (*using tables*), all values are close to or above 0.4. In general, the fit of the model was acceptable, but not perfect (RMSEA = 0.092; 90%-confidence interval 0.081–0.103; AGFI = 0.866).

Taking into consideration the loadings for item 5 (*homework*) on both scales, observed in exploratory factor analysis, an alternative structural model was tested with a path also from the *Learning* latent variable to this item. This is also justified from a theoretical point of view. Being given difficult homework involves both a learning situation and elements of being tested when the work is checked, usually in front of the class. This model had a more satisfactory fit (RMSEA = 0.075; 90%-confidence interval 0.064–0.087; AGFI = 0.905), suggesting that this item contributes to both factors. In the modified model, paths to this item were 0.42 and 0.39 for *Learning* and *Testing* scales respectively and the correlation between the latent variables decreased to 0.63.

### AMAS criterion validity

As was stated in the predictions section, several correlational analyses were conducted in order to examine the convergent and discriminant validity of the AMAS. All respective data is presented in Table 2.

**Table 2:** **Convergent and discriminant validity of the AMAS questionnaire**.

**Group**	**Measure**	***n***	**AMAS total**	**AMAS Learning**	**AMAS Testing**
Self assessed math skill (MAAA)	Math skill	809	−0.50^**^	−0.36^**^	−0.49^**^
	Arithmetic skill	808	−0.47^**^	−0.36^**^	−0.45^**^
	Geometry skill	809	−0.43^**^	−0.34^**^	−0.41^**^
	Text problems skill	808	−0.44^**^	−0.31^**^	−0.44^**^
Self report of math scores during education (MAAA)	Typical grade–elementary school	809	−0.32^**^	−0.30^**^	−0.26^**^
	Typical grade–gymnasium	798	−0.39^**^	−0.32^**^	−0.35^**^
	Typical grade–high school	809	−0.38^**^	−0.28^**^	−0.36^**^
	Typical grade–average	810	−0.44^**^	−0.36^**^	−0.40^**^
Discouragement when solving problems (MAAA)	Discouragement–Math problem	810	−0.48^**^	−0.37^**^	−0.46^**^
	Discouragement–Essay	809	0.09^*^	0.05	0.10^**^
Non-allowed help usage (MAAA)	Non-allowed help–Math	808	−0.46^**^	−0.36^**^	−0.43^**^
	Non-allowed help–Humanities	802	0.06	0.03	0.07
Liking school subjects (MAAA)	I like Math	809	−0.50^**^	−0.36^**^	−0.50^**^
	I like Sciences	809	−0.32^**^	−0.23^**^	−0.32^**^
	I like Humanities	808	0.12^**^	0.08^*^	0.12^**^
Temperament (FCB-TI)	Sensory Sensitivity	130	0.13	0.15	0.09
	Emotional Reactivity	130	0.48^**^	0.35^**^	0.48^**^
	Perseverance	130	0.28^**^	0.21^*^	0.28^**^
	Activity	130	−0.03	0.03	−0.07
	Briskness	130	−0.27^**^	−0.16	−0.31^**^
	Endurance	130	−0.27^**^	−0.15	−0.30^**^
Anxiety (STAI)	State Anxiety	280	0.22^**^	0.18^**^	0.20^**^
	Trait anxiety	818	0.33^**^	0.22^**^	0.34^**^

*Names of measurement instruments used are presented in parentheses in the first column. All correlations are reported with the respective sample size, which differs considerably in several cases. Significant correlations are marked with asterisks*.

** *p < 0.01 (two tailed)*;

* *p < 0.05 (two tailed)*.

For clarity of description we only present a simplified correlation matrix, in which only correlations between the AMAS scores with external measures are presented. The complete correlation matrix is presented in Data Sheet 2.

As can be seen in Table 2, the AMAS scores strongly correlated with self-assessed math skills: higher levels of math anxiety were associated with poorer self-assessed math competence. Visual inspection of scatterplots representing the relationship between the AMAS total score and average school grade and the AMAS total score and self-assessed math skill showed no departures from a linear relationship. It was also corroborated by inspection of the Lowess curves superimposed over the scatterplots. The same was true in case of both AMAS scales.

This negative correlation is present for all fields of math included in the scale. Interestingly, the relation was stronger for math skills in general than for geometry (*p* < 0.001). The AMAS scores correlated negatively with self-reported typical math scores at all levels of education. Participants with a higher level of math anxiety achieved worse grades (in the Polish system of school grades, numerically high grades correspond to good scores). Interestingly, when the correlation between the AMAS and self-assessed math skills was compared to the correlation between the AMAS and average school grade, the latter was significantly lower (*p* = 0.015). Hence, math anxiety is more strongly related to self-assessed skill than to school grades (but it correlates with both).

Moreover, participants showing higher levels of math anxiety reported getting discouraged faster when struggling with math problems. Interestingly, in the case of struggling with difficult essays, they perceived themselves to be more persistent. Here, the correlation with the AMAS was very small but positive. This correlation may be caused by several factors—highly math anxious participants prefer humanities because of better performance in the latter. On the other hand, participants might simply have contrasted their persistence in math and humanities and the latter seemed much higher to them. Higher math anxiety was associated with more use of non-allowed aids when struggling with math problems, but did not correlate with it in the case of humanities (all based on self-reports). Higher math anxiety was associated with less liking of math and science, but the correlation was significantly smaller in the case of science (*p* < 0.001). Contrarily, higher math anxiety was associated with more liking of humanities. Therefore, the AMAS score can neither be accounted for by general attitude toward school and school subjects nor by lack of persistence when struggling with problems. Math anxiety is specifically negatively related to math skills (objectively and self-assessed) and to math attitudes. Contrarily it is not correlated (or sometimes positively correlated) with all these factors with regard to humanities.

The AMAS correlations with temperamental traits revealed an interesting pattern of results. Temperament as an elementary characteristic should be considered as primary to math anxiety and some attempts at explaining math anxiety may be based on temperamental traits. Math anxiety did not correlate with *Sensory Sensitivity* or *Activity*. A high positive correlation with *Emotional Reactivity* may be interpreted as an indication that math anxiety may be a form of an exaggerated emotional response toward math problems. On the other hand, a moderately positive correlation with *Perseverance* may suggest that math anxiety is increased by mentally elaborating too long about unsuccessful attempts to deal with the problem. A moderately negative correlation with *Endurance* and with *Briskness* may indicate that math anxiety is low in participants whose behavior can be described as highly energetic and persistent.

Interestingly, correlations with state and trait anxiety were moderate. This indicates that math anxiety cannot be accounted for by anxiety in general. Moreover, as predicted, correlations of math anxiety with state anxiety were numerically smaller than those with trait anxiety. Nevertheless, this difference in correlations did not reach significance (*p* = 0.150).

In the subsequent step we tested whether correlations between AMAS, MAAA, and Anxiety measures differ between the math and the non-math group. Surprisingly, virtually all correlations of AMAS scores and MAAA were significantly different from zero only in the non-math group. In the math group correlations with math-related items were smaller than 0.20 and in the large majority of cases not significantly different from zero. Only correlations with state and trait anxiety were significantly larger than zero. This effect was not caused by reduced variance e.g., because of floor or ceiling effects.

To further explore gender differences in AMAS scores we tested its correlations with external measures for female and male participants separately. As regards MAAA, correlations were stronger for female participants. In male participants correlations of *Learning* scale were null and non-significant. For *total* score and *Testing* scale correlations were smaller but significant. Reverse pattern of correlations was observed in case of state and trait anxiety measures. Its relation to AMAS scores were more pronounced in male participants.

**Figure 3:**
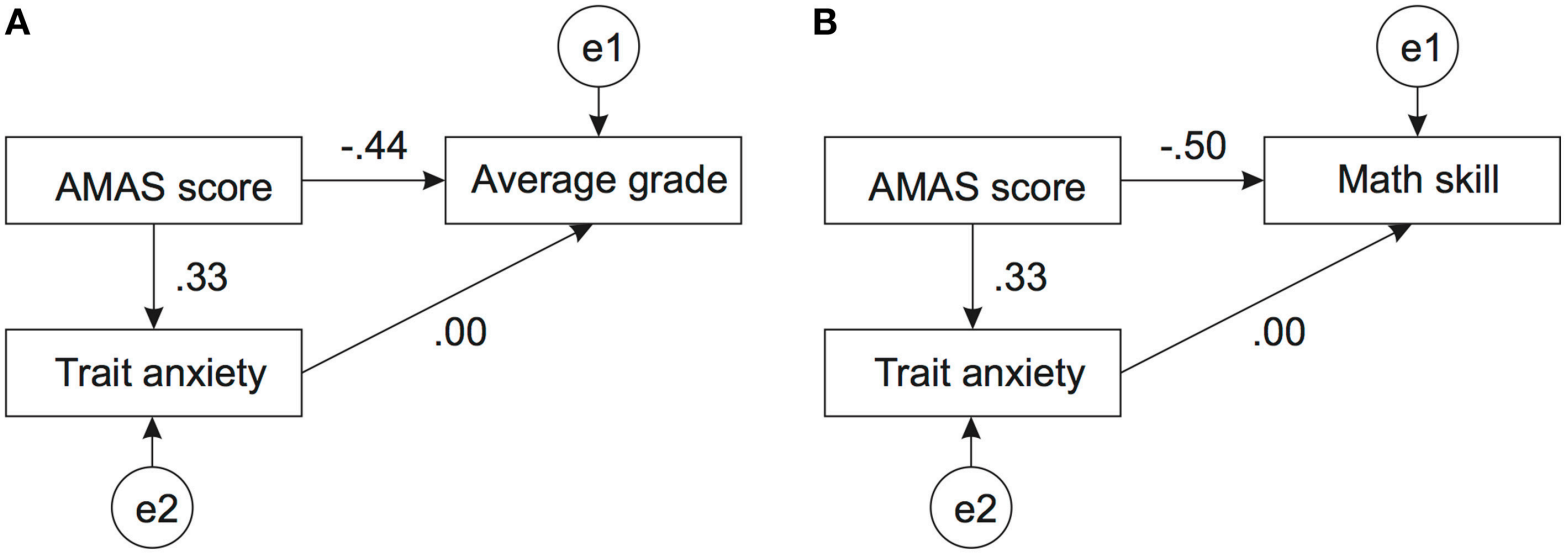
**Path model of the relation between trait anxiety, AMAS score, and math ability**. Panel **(A)** depicts the relation between these two variables and the average math grade. Panel **(B)** depicts the analogous relation with self-assessed math skill. Both models reached satisfactory fit only when the relation between trait anxiety and the math ability measure was set to zero. All other coefficients were significantly different from zero. Variables labeled with e1, e2 etc…denote the respective error terms.

In order to further explore relations between math anxiety, trait anxiety, and math skills (both grades and self-assessed skills) we performed path analyses. The first path analysis comprised relations between AMAS, trait anxiety and school grades. The path model together with standardized coefficients is presented in Figure 3A. The model reached satisfactory fit (RMSEA = 0.027) only when the path between trait anxiety and grades was set to 0. All depicted coefficients were significantly different from zero.

Assuming a possible relation between Math anxiety, trait anxiety and self-assessed math skill, the fit of the path model was worse, but still acceptable (RMSEA = 0.094), only when the relation between trait anxiety and math skill was fixed at 0. The path model together with the standardized estimates is presented in Figure 3B. All estimates were significantly different from zero. Henceforth, we can conclude that there is a specific relation between math anxiety and math performance, which cannot be accounted for by general anxiety.

### Similarities and differences between results of AMAS between American, Italian, Iranian, and Polish samples

In the last step of the analysis we examined whether the results obtained in our study resembled those reported in a study by Hopko et al. (2003) as well as Iranian (Vahedi and Farrokhi, 2011) and Italian (Primi et al., 2014) AMAS adaptations. The respective data are presented in Table 3.

**Table 3:** **Comparison of the AMAS questionnaire results between the Polish (presented here), US-American (Hopko et al., 2003), Italian (Primi et al., 2014), and Iranian (Vahedi and Farrokhi, 2011) samples**.

**Measure**	**Polish sample**	**American: development sample**	**American: replication sample**	**Italian sample (college students)**	**Iranian sample**
Mean Score (SD)	21.9 (6.6)	21.9 (7.0)	23.2 (5.8)	21.6 (6.3)	18.4 (6.8)^#^
Mean Score–Female participants (SD)	22.6 (6.6)	21.9 (6.9)	23.8 (5.7)	22.1 (6.0)	n.a.
Mean Score–Male participants (SD)	18.8 (6.7)	19.5 (6.9)	21.5 (5.7)	20.8 (6.6)	n.a.
Correlation between subscales^+^	0.49 (0.44–0.54)	0.62 (0.53–0.70)	n.a.	n.a.	0.50 (0.41–0.58)
Correlation *Learning*-total	0.85 (0.83–0.87)	0.88 (0.84–0.91)	n.a.	n.a.	0.85 (0.81–0.88)
Correlation *Testing*-total	0.88 (0.86–0.89)	0.91 (0.88–0.93)	n.a.	n.a	0.88 (0.85–0.90)
Cronbach Alpha–total	0.85 (0.83–0.86)	0.90 (0.88–0.92)	0.83(0.79–86)	0.85 (0.82–0.88)	0.82 (0.79–0.85)
Cronbach Alpha–*Learning*	0.78 (0.76–0.80)	0.85 (0.81–0.88)	0.74 (0.68–0.79)	0.80 (0.76–0.84)	0.75 (0.70–0.79)
Cronbach Alpha–*Testing*	0.84 (0.82–0.86)	0.88 (0.85–0.90)	0.81 (0.77–0.85)	0.83 (0.80–0.86)	0.79 (0.75–0.83)
Test-retest reliability total^*^	0.71 (0.67–0.74)	0.85 (0.81–0.88)	n.a.	n.a.	n.a.
Test-retest reliability Learning^*^	0.59 (0.45–0.70)	0.78 (0.72–0.83)	n.a.	n.a.	n.a.
Test-retest reliability Testing^*^	0.71 (0.60–0.79)	0.83 (0.78–0.89)	n.a.	n.a.	n.a.
Correlation AMAS and math grades	−0.44 (−0.49 to −0.38)	−0.52 (−0.61 to −0.41)	−0.34 (−0.45 to −0.22)	n.a.	n.a.

*Several characteristics are similar. For further details please refer to the main text*.

# *Inspection of the results from the Iranian study suggests that responses were coded from 0 to 4 instead of 1–5, therefore the average score reported here was obtained by adding nine to the average score reported in the original paper (see Table 2 there)*.

* *For the Polish sample the test-retest delay was 4 months whereas for the US-American sample it was 2 weeks*.

+ *Numbers in parentheses after correlation/reliability estimate indicate 95%-confidence intervals*.

As far as descriptive statistics are concerned, the results in all countries are very similar. Unfortunately, psychometric properties and statistics were not provided in all studies. In general, all other correlations are rather similar across the different language versions.

Correlations between the AMAS score and state and trait anxiety in the Polish and American sample were similar. Nevertheless, in case of the Polish sample, the correlation with trait anxiety was higher. Interestingly, the observed correlation between math anxiety and math achievement (self-reported, based on typical school grades) was stronger than the estimated population correlation between math anxiety and math achievement reported in a metaanalysis by Ma (1999). This may be due to differences in the measurement of math skills. Because such high correlations between math achievement and math anxiety in Poland were already found in the PISA study (see Lee, 2009), the current study points to culture-specific variations of validity of the AMAS.

## Discussion

### Overview

Usefulness of the Polish version of the AMAS questionnaire was studied in a large sample of Polish adults. We observed few differences between cultures, but confirmed previously reported gender differences. Good psychometric properties (both validity and reliability) of the Polish version suggest the usefulness of the AMAS in another cultural and linguistic context that is somewhat different from those that were already tested, namely in an Eastern Europe culture.

### AMAS reliability

The AMAS is characterized by very good reliability properties as assessed by both Cronbach alpha as well as test-retest correlation. When the ordinal alpha coefficient, considered to be more suitable for the Likert scale response format (see Zumbo et al., 2007), was computed, the reliability estimates were even numerically higher. In our study, the 4-months period between initial testing and retest was quite long compared to typical test-retest reliability study designs, which usually encompass only a few weeks. Nevertheless, satisfactory test-retest estimates indicate that math anxiety is substantially stable over time. When interpreting the values, one must keep in mind that the retest sample was very homogeneous, comprising only psychology students. Therefore, reliability might be even higher for the general population.

### AMAS validity

#### Construct validity

The factor structure obtained in the Polish sample supports a two-factor solution, one factor referring to math learning anxiety and the other to math testing anxiety. Based on our analysis, the item concerning being given difficult math homework should not be included in the *Learning* scale in our Polish sample. Factor loadings for this item were very similar for both factors. Double loadings are different from the original sample, but in our view not inconsistent with item content, because it refers both to learning and being exposed to evaluation afterwards. Normally, items with double loadings are excluded. However, this item is characterized by a strong item-total correlation and therefore, it would not be recommended to exclude it from the scale.

#### Convergent and discriminant validity–general measures

The results of the convergent and discriminant validity analyses also revealed satisfactory results. As expected, the AMAS scores correlated moderately with state and trait anxiety, a trait measure for *Negative Emotionality*, a trait measure of *Perseverance*, and trait measure for *Endurance*. Highly math anxious individuals are somewhat more state- and trait-anxious in general, are more likely to respond with negative emotions in a wide range of situations, and have lower general endurance. The latter correlation is in line with the observation of local avoidance observed in highly math anxious individuals. When facing a math problem, these individuals tend to terminate the anxiety-evoking situation by impulsively providing the answer and not considering its accuracy (Ashcraft and Ridley, 2005). The correlations with other temperament trait measures were null or did not significantly deviate from zero, which may be taken as evidence for discriminant validity of the AMAS. Henceforth, we conclude that the AMAS is related to some general psychological characteristics. Nevertheless, the generally moderate correlations in a large sample suggest that math anxiety is a unique trait that cannot be reduced to or fully explained by those general traits discussed above. All these correlations hold irrespective of whether participants study math related or math unrelated subjects.

#### Convergent and discriminant validity–math related measures

Indications for both, convergent and discriminant validity of the AMAS, were observed. The AMAS correlated negatively with self-assessed math skill, but the correlation between the AMAS score and self-assessed math skill in general was significantly larger than the correlation with self-assessed geometry skill. This is in line with results obtained in children by Vukovic et al. (2013), suggesting that math anxiety is more related to mathematical operations using abstract symbolic material.

The AMAS score also correlated with self-reported math grades at all levels of education. Furthermore, consistent with previous US-American studies, the correlation between self-assessed math skills was more pronounced than the correlation between math anxiety and (self-reported) school grades (Ashcraft and Ridley, 2005).

However, the relationship observed in our study is considerably stronger than the average correlation between math anxiety and math achievement (see: Ma, 1999). One must also keep in mind that we used self-reported math grades instead of official school documentation. However, it was shown that these measures are valid in US-American participants (as regards SAT score; see Nosek et al., 2002). What is more, the results of the PISA 2003 study suggest that in Poland the relationship between math anxiety and math achievement is above average (Lee, 2009). This literature suggests that the stronger relationship between math anxiety and math achievement in Poland may be real and not an artifact of the self-assessment question. Nevertheless, to be sure, this has to be examined in future studies.

Highly math anxious participants also reported getting discouraged more easily when facing difficult math problems, but not when writing an essay. This kind of behavior resembles the mechanism of local avoidance already described above. Highly math anxious participants also reported using more non-allowed aids than low anxious participants when solving math problems, but not when solving problems in humanities. Furthermore, highly anxious individuals also reported liking math less than low anxious individuals. This was more pronounced than the relationship between the AMAS score and liking science.

In sum, highly math anxious individuals report worse math performance and more specific negative attitudes toward math. However, this correlation pattern was present only in individuals from non-math group (i.e., those who study math unrelated subjects). In the math group we did not observe correlations between math related measures and math anxiety. This result deserves more attention in future studies.

### Comparison of four language versions of AMAS

In general, both average scores as well as important psychometrical properties of the AMAS were very similar for the US-American, Italian, Iranian and the Polish versions. Results of Polish version fall between results from other versions as regards average scores, correlations and reliability estimates. The only substantial difference was a lower test-retest reliability estimate of the *Learning* scale in Polish than in the US American sample (which is the only for which such reliability estimates are available). This is not necessarily due to a cross-cultural difference, because the Polish retest sample was very homogeneous and the test-retest interval was much longer—therefore lower reliability scores are to be expected. Furthermore, in the US American sample the subscales were more strongly correlated than in Polish sample, unfortunately such estimates were not provided in Italian and Iranian studies.

### Gender differences in math anxiety

The factor structure of AMAS was very similar for male and female participants (see note in Data Sheet 1). In our study we found a significant mean difference in math anxiety between male and female participants. This is in line with several studies conducted up to date. The estimated effect size can be considered middle sized (*d* = 0.61). This effect is stronger than the estimate provided by Hembree (1990) in his meta-analysis (*d* = 0.31). Interestingly, effect size of gender difference for all OECD countries PISA 2012 (OECD, 2013) study is almost the same (*d* = 0.30). As PISA study shows, this gender difference in Polish adolescents is very small (*d* = 0.11). Such discrepancy in estimated effect size of the gender difference may originate from the fact that participants of PISA study were adolescents (15-year-olds) whereas we tested students. The other reason may be that different instruments were used to measure math anxiety.

Interestingly, the observed gender difference was largely driven by *Testing* scale. However, it requires further investigation whether larger effect size in case of *Testing* scale originates from the fact that strong floor effect was observed in case of *Learning* scale. The other explanation could be that *Testing* scale may be more strongly related with test anxiety. It was shown that, contrarily to male, female individuals perceive testing situation as threat rather than challenge (Zeidner, 1998). Gender differences in test anxiety are most pronounced in its emotional aspect, and this pattern of results, together with higher test anxiety levels in female individuals is present in numerous and varied cultural and linguistic contexts (Zeidner, 1998). Our results thus suggest that gender differences in math anxiety may be modulated by test anxiety. This aspect deserves attention in further research. Interestingly, correlations between AMAS scores and math related measures were more pronounced in female participants whereas in case of state and trait anxiety this pattern was reversed. This effect deserves further attention in future studies. Furthermore, the observed gender difference may be partly driven by differences depending on the field of study, because we observed few significant correlations with math-related measures in participants with higher math expertise. The bigger gender difference in math anxiety observed in our study may be caused by the fact that the group of education students, which was very high in math anxiety—in line with American studies (see: Hembree, 1990)—was mostly comprised of female participants.

Explanations of such gender differences in math anxiety mostly refer to socio-cultural factors (Devine et al., 2012). Namely, male individuals are discouraged from expressing their anxiety. On the other hand, they are also expected to perform better in math.

### Limitations of the presented study

One must keep in mind that our study is mostly based on self-reports. At first glance, this is self-explanatory when considering the nature of the constructs investigated. However, we argue that in light of the detailed results and the available literature, this does not undermine our conclusions in general, because for instance, the validity of self-reported math grades has previously been shown (Nosek et al., 2002).

Nevertheless, we have to admit that some of our results must be taken with some caution. First, participants provided their typical grades in math at several stages of their regular education.

Therefore, their reported grades had been given several years ago, possibly biasing the grades reported. One may also argue that it would be better to ask about scores in standardized math achievement tests. However, we believe that here, because of specificity of Polish educational system such questions would not be valid. First of all, standardized achievement tests are administered three times: after elementary school, after secondary school and after high school. Nevertheless, first two exams do not comprise math as a separate subject but it is a part of “science” module. Furthermore, in the exam after high school math was not compulsory for several years, so many of our participants could not have taken it. Second, the exam scores are used mostly for the purpose of next educational level applications and people usually do not remember how many points they scored. Furthermore, the responses observed for items from the MAAA scale were given at the same time, and the content of the items was similar. It is easily possible that the correlation between responses to several items may to some extent have been driven by this similarity. Moreover, attitudes toward humanities may be rated by contrasting them to responses to items referring to math and science. Future studies should address these issues, for instance by constructing more complex and psychometrically validated scales of the respective attitudes. This is especially the case for the MAAA scale, because its items were constructed in such a way that responses to each item were considered as separate (each item referring to one aspect). No calculation of composite scores was possible (apart from the average math grade obtained by averaging math grades from all three stages of math education). As a consequence it was impossible to conduct a psychometric evaluation of this scale.

Finally, objective measures of math ability and achievement should be utilized. This is of particular importance since the relationship between math anxiety and performance differs considerably when different measures of achievement are involved, as reported in the meta-analysis by Ma (1999). The relationship is stronger when teacher assessments as well as research methods are used in order to measure performance. The magnitude of this relationship is smaller when standardized achievement tests are utilized (Ma, 1999). All these factors may have increased some correlations with other constructs.

However, there is also one limitation that may have led to a decrease in correlations or even may make the overall picture of math anxiety too simplified. Our study sample was only comprised of university students and was homogeneous as regards age and educational level (as expressed by total number of years of education). However, it has shown considerable differences in correlations of math anxiety depending on field of study. Henceforth, further research should test more heterogeneous samples (elderly people, adolescents, individuals who have no educational experiences at universities). This is of particular importance in order to extrapolate our conclusions to the general population.

In sum, the general pattern of correlations is consistent with the literature and the assessment of traits and performance used here has generally shown to be valid in previous studies. Therefore, we are confident that the general pattern of results, which is largely consistent with the pattern of other samples, is valid. However, it is possible that the level of the observed correlations and effect sizes will differ for other sample characteristics, assessment tools or assessment procedures. All these questions deserve further investigation.

## Overall conclusions

Based on the results of our study, we conclude that the AMAS scale in the present form can be used for the Polish population. More interestingly, the presented study provides further support for the claim that the math anxiety construct might be generalized across many cultures. Gender differences were confirmed in the present study; they were even a bit larger than reported so far. Keeping in mind the importance of math education, extensive research should be conducted in countries from several continents in order to develop adequate tools to measure math anxiety or to examine whether assessment tools developed in one country can be used in another. In case of the AMAS, our data suggests that this is the case.

## Author contributions

KC and MS designed the study, collected the data and prepared the data for analysis. KC, HCN, and KW analyzed the data. KC, MS, HCN, and KW wrote the manuscript and accepted its final version.

### Conflict of interest statement

The authors declare that the research was conducted in the absence of any commercial or financial relationships that could be construed as a potential conflict of interest.
