# Supplementary material for: Math Anxiety Assessment with the Abbreviated Math Anxiety Scale: Applicability and Usefulness: Insights from the Polish Adaptation
Source: Front Psychol. 2015 Nov 30;6:1833. doi: 10.3389/fpsyg.2015.01833 (PMC4663255; doi:10.3389/fpsyg.2015.01833)
Supplement: Supplementary file 1 [file DataSheet1.DOCX]

Cipora, K., Szczygiel, M., Willmes, K., Nuerk, H-C. (2015). Math Anxiety Assessment with the Abbreviated Math Anxiety Scale: Applicability and usefulness: insights from Polish adaptation. *Front Psychol. 6:*1833. doi: 10.3389/fpsyg.2015.01833

**Appendix A – Exploratory factor analysis**

In order to examine the internal structure of the AMAS, an exploratory principal axes factor analysis (PFA) was used. This extraction method is more suitable than the more popular principal component analysis (PCA) when the multivariate normal distribution assumption is violated. The data was suitable for an exploratory factor analysis according to different criteria (KMO = .86; Bartlett test for sphericity significant at *p* < .001). Based on the scree plot, a two-factor solution was found which accounted for 61.6% of the variance (*first* component 44.8 % and *second* component 16.8%). As it was postulated theoretically that components of math anxiety are correlated, an oblique rotation was applied (simple OBLIMIN). The factors obtained in the analysis refer to two components of math anxiety - anxiety related to learning math and being evaluated in math. Factor loadings from the model matrix are presented in Table A.

To make the analysis more comparable to the analysis performed by Hopko et al (2003), we additionally performed an exploratory factor analysis with the principal component (PCA) extraction method and the orthogonal VARIMAX rotation as well. Total variance explained was the same as in case of oblique rotation. However, in case of orthogonal rotation, the first factor referred to math testing and the second to math learning. Factor loadings for the VARIMAX rotation are presented in the right part of Table A.

The analysis was conducted for female and male participants separately and in both instances it revealed two factor solution with very similar factor loadings as well as amount of variance explained.

Table A. Factor loadings for exploratory factor analyses with oblique and orthogonal rotations.

| Item | Item description | Scale | Factor loadings (OBLIMIN rotation) | | Factor loadings, VARIMAX rotation | |
| --- | --- | --- | --- | --- | --- | --- |
|  |  |  | 1 (*Learning*) | 2 (*Testing*) | 1 (*Testing*) | 2 (*Learning*) |
| 1 | Using tables | *Learning* | .40 | -.12 | .21 | .53 |
| 2 | Test one day before | *Testing* | .08 | -.79 | .83 | .24 |
| 3 | Watching teacher’s work | *Learning* | .65 | -.08 | .22 | .73 |
| 4 | Math exam | *Testing* | -.13 | -.88 | .88 | .04 |
| 5 | Homework | *Testing* | .32 | -.50 | .64 | .42 |
| 6 | Attending lecture | *Learning* | .83 | .09 | .11 | .82 |
| 7 | Other student explaining Math | *Learning* | .76 | .12 | .03 | .80 |
| 8 | Pop quiz | *Testing* | .05 | -.72 | .81 | .17 |
| 9 | New chapter | *Learning* | .51 | -.24 | .36 | .61 |
